# Cultural Adaptation and Development of an Educational Intervention *‘Meri Sehat, Mere Rules’* Relating to Cardiovascular Disease Associated with Rheumatoid Arthritis for South Asian People

**DOI:** 10.31138/mjr.120623.ado

**Published:** 2024-06-30

**Authors:** Ruman Tiwana, Atiya Kamal, Dilsher Singh, Durga Prasanna Misra, Afshan Salim, Faika Usman, Holly John, George D. Kitas, Sheila Greenfield, Prem Kumar, Claire Ray, Ailsa Bosworth, Ayesh Ahmad, Joti Reehal, Kanta Kumar

**Affiliations:** 1 Institute of Clinical Sciences, College of Medical and Dental Sciences, University of Birmingham, United Kingdom,; 2 School of Social Sciences, Birmingham City University, United Kingdom,; 3 Clinical Lead Community Cardiology Service, ENKI Medical Practice, United Kingdom,; 4 Clinical Immunology and Rheumatology, Sanjay Gandhi Postgraduate Institute of Medical Sciences (SGPGIMS), Lucknow, India,; 5 Bellevue Medical Centre, Birmingham, United Kingdom,; 6 Department of Rheumatology, Dudley Group of Hospitals NHS Trust, Dudley, United Kingdom,; 7 Institute of Applied Health Research, College of Medical and Dental Sciences, University of Birmingham, United Kingdom,; 8 School of Biomedical Sciences, Institute of Clinical Sciences, College of Medical and Dental Sciences, University of Birmingham, United Kingdom,; 9 National Patient Champion for National Rheumatoid Arthritis Society (NRAS), United Kingdom,; 10 Patient Partner, NRAS, United Kingdom,; 11 Royal Wolverhampton NHS Trust, Wolverhampton, United Kingdom

**Keywords:** rheumatoid arthritis, cardiovascular disease, educational intervention, culturally adapted, South Asians

## Abstract

**Background:**

The cardiovascular disease (CVD) risk is elevated by 1.5 times among South Asians with rheumatological conditions like rheumatoid arthritis (RA) in the UK. However, there is a dearth of culturally sensitive educational interventions tailored to this population. We have culturally adapted an existing cognitive behavioural patient education intervention, originally designed for predominantly White populations, to address this gap.

**Methods:**

The adaptation process followed the Ecological Validity Model, comprising four phases: stage-setting and expert consultations, preliminary content adaptation, iterative content adaptation with patient partners, and finalisation with patient partners and feedback. The Theoretical Domains Framework (TDF) was employed to evaluate the relevance, acceptability, and cultural adaptation of the existing intervention. Seven South Asian Patient Experts with RA were interviewed, and their input aided in developing new content for the culturally sensitive intervention.

**Results:**

The intervention was successfully adapted to suit South Asians. Cultural adaptation involved reviewing elements of the existing intervention, including language tone, content, and metaphors. Moreover, by incorporating behaviour change techniques, the content was designed to enhance understanding of RA, CVD risk associated with RA, and promote a healthy lifestyle. The newly developed educational intervention addressed topics such as community resistance, perspectives on health and culture, societal pressure, and opportunities for change. Key messages were visually illustrated through pictorial diagrams in a twenty-five-minute online resource.

**Conclusion:**

The first culturally adapted CVD intervention targeting South Asian individuals with RA, particularly those who are non-English-speaking, is now accessible free of charge at www.nras.org.uk/apnijung nationally and internationally.

## INTRODUCTION

Age, gender, ethnicity, hypertension, diabetes, smoking, and hyperlipidaemia are established risk factors for cardiovascular disease (CVD).^1^ However, in the last two decades, emerging evidence has identified chronic inflammation as a non-traditional risk factor for increased CVD risk.^2^ Rheumatoid arthritis (RA) serves as an illustrative example.^3^ RA, affecting approximately 1% of the general population, is a global condition characterised by polyarthritis, progressive joint damage, immunological abnormalities, systemic inflammation, heightened morbidity, and premature mortality.^2^ It is now well-established that CVD is a common comorbidity in RA patients.^2^ Notably, atherosclerosis severity and burden are found to be elevated in various minority ethnic populations, including South Asians.^3^ RA patients of South Asian origin also bear a significant CVD burden.^4,5,6^

International studies have highlighted that a standardised approach to interventions or treatment plans does not adequately address modifiable factors, leading to challenges in improving health outcomes and potentially exacerbating health inequalities.^4,5^ People with RA generally have poor knowledge regarding CVD risk, as patient education in rheumatology clinics often overlooks this aspect.^7^ Furthermore, individuals of South Asian background living with RA in the UK exhibit even lower CVD risk knowledge compared to White populations.^8^ This disparity may stem from interventions primarily targeting White populations, thereby limiting access to educational knowledge and perpetuating health disparities.^8^ The “one size fits all” approach is problematic and accentuates health inequalities, resulting in suboptimal health outcomes.^8^ Culturally sensitive interventions that accommodate diverse patient groups are crucial in addressing this issue, considering the increasingly diverse population in the UK and globally.^9,10^ Studies recommends understanding the cultural context and drivers influencing the adoption of protective health behaviours within social networks to develop culturally focused interventions.^9,10^

There are examples of understanding the cultural context and drivers in interventions (for example, for cancer, asthma, mental illness, and diabetes),^10^ that emphasise the integration of cultural knowledge can improve health outcomes among people living with chronic disease. Emerging data, predominately from the diabetes literature, suggests changes to people’s health protective behaviour increases after culturally adapted interventions are offered,^8^ Netto et al.^10^ propose that cultural adaptations should aim to create a culturally equivalent version of a prevention programme, ensuring that elements of the original intervention are revised to meet the needs of people from a diverse and inform cultural beliefs appropriately. They outline steps to guide the decision-making process for culturally adapting evidence-based interventions. ^10^

Previous studies where such cultural adaption has taken place offer an encouraging platform as they demonstrate that educational interventions that incorporate cultural drivers of behaviour to include knowledge, awareness and risk perception are likely to be more effective than interventions that are not culturally sensitive.^11^ Hence, the importance to learn from existing culturally sensitive interventions that address CVD risk knowledge among those from minority ethnic origins as well as considering the local context needs of people from a South Asian origin.^10^ In addition, there is evidence that interventions that are informed by staged process are likely to capture the essence of the adaptations. Thus, we used the Ecological Validity Model, comprising four phases.^12^ There is wealth of evidence that highlights embedding theory within interventions is key to encourage behavioural change, to this end we used Theoretical Domains Framework (TDF) to adapt the existing CVD intervention.^13,14^ The existing CVD intervention developed by John et al.^4^ for White British patients living with RA also utilised the TDF framework to target behaviour change. While originally designed for the White population, John et al.^15^ intervention serves as a foundational platform from which we can adapt and tailor the content to better suit the cultural needs of the South Asian population. The aim of this study was to culturally adapt the cognitive-behavioural patient education intervention developed by John et al.^15^

## METHODS

The study adhered to the SQUIRE guidelines (https://www.equator-network.org/reporting-guidelines/squire/) when reporting the findings. The cultural adaptation of the cognitive-behavioural patient education intervention followed the Ecological Validity Model,^12^ involving four phases: 1. Stage setting and expert consultations; 2. Preliminary content adaptation; 3. Iterative content adaptation with patient partners; 4. Finalised adaptation with patient partners and feedback. The Theoretical Domains Framework (TDF),^13^ was utilised to assess the relevance, acceptability, and cultural content adaptation of the intervention. The development of the intervention involved a multidisciplinary steering group comprising patient partners, rheumatologists, cardiology experts, general practitioners, a health psychologist, physiologists, and a medical sociologist, all working collaboratively as the research team.

### Stage setting and expert consultations

Seven participants (aged >18) diagnosed with RA were recruited for the study, comprising six females and one male. Recruitment was conducted through the National Rheumatoid Arthritis Society (NRAS) (n=5) and the community (n=2), with a specific focus on individuals from a South Asian background. All participants had a good understanding of South Asian culture and traditions and were proficient in English. Participants, who were employed and had family/societal responsibilities, provided informed consent prior to their participation. Communication about the study was initiated via email, followed by a telephone conversation to address queries and schedule an online Zoom session at a mutually convenient time.

Two semi-structured interview guides were developed by RT and AK and JR which were informed by the TDF and by the work of John et al.^15^ ‘heart disease in Rheumatoid Arthritis Patient Manual’. **Table 1** outlines topics explored during patient partner interview sessions 1 and 2. Questions were mapped onto each of the TDF domains to explore participants’ perceptions of: (1) How well participants understood the existing cognitive behavioural intervention, (2) how they would feel while using it, (3) how well it served its purpose, and (4) how it aligned with their health, illness and cultural beliefs.

**Table 1.** Topics explored during patient partners’ interviews mapped onto the theoretical domains’ framework.

**Topics discussed with participants: Session 1**	**Topics discussed with participants: Session 2**
Knowledge: The overall content discussed in the manual; do you think that people of a South Asian background know this? Do you think people of a South Asian background know they should be doing this? Do you think people of a South Asian background know why they should be doing this? What do you think about the content of the manual, ie, the examples, scenarios and images included within the manual?	Knowledge: Have you come across either the National Rheumatoid Arthritis Society (NRAS) or Apni Jung (‘Our Fight’) webpage? Do you think a South Asian person with Rheumatoid Arthritis would know where to access the video? Do you think a South Asian person with Rheumatoid Arthritis would know how to access the video? Can you think of any situation(s) why a South Asian person would decide not to access the video?
Skills: Do you think a South Asian person would have the skills (i.e., the ability to do something) to follow the content and activities in the manual? Do you think the content and activities included in the manual would allow South Asian people to develop the skills required to follow the manual?	Skills: Do you think a South Asian person would have the skills required to access the video? How easy or difficult do you think it would be for a South Asian person to follow the video?
Social/Professional Role and Identity: Do you think what is included in the manual can be carried out by a person from a South Asian background? Do you think what is included in the manual can be carried out by a person from a South Asian background?	Social/Professional Role and Identity: Do you think completing an online video is in line with the identity of South Asian people?
Beliefs about Capability: How confident do you think people of a South Asian background may feel using the manual? How confident do you think people of a South Asian background may feel in following the scenarios? How confident do you think people of a South Asian background may feel doing the homework set? How confident do you think people of a South Asian background may feel doing the activities? What do you think would make a person of a South Asian background more confident to using the manual?	Beliefs about Capability: How easy or difficult do you think a person from a South Asian background would find it to access the video?
Beliefs about Consequences: Having had a look at the manual. What do you think are the key messages within the manual? (i.e., what do you think works very well and what do you think doesn’t work very well). To what extent do you believe that the manual will help patients from a South Asian background? (i.e., what are the benefits of the manual, how can the manual help, what are the risks of the manual). Is it important that people of a South Asian background use the manual? What do you think the consequences are if a person from a South Asian background does not follow the manual?	Beliefs about Consequences accessing the online manual: To what extent do you believe that an online training resource will help a South Asian person with Rheumatoid Arthritis? To what extent do you believe referring people with Rheumatoid Arthritis via clinics to this resource will be helpful?
Social Influences (Norms): Does the content of the manual contain conflicting beliefs that may be encouraged by people from a South Asian background when managing Rheumatoid Arthritis and the increased risk of heart disease. Does the manual reflect the social influences from family and/or friends that South Asian patients are likely to encounter? Can you suggest anything else that could be added or changed to the manual to reflect family and/or friends’ influence on managing the increased risk of heart disease. Does the manual reflect cultural influences that South Asian patients are likely to encounter?	Social Influences (Norms): Do you think a South Asian person, if they are to follow the video would have the support of others to do it?
Motivations and Goals: Do you think the goals related content within the manual are realistic for people of a South Asian background (ie, weight, diet, and exercise)?	Motivations and Goals: Would it be a priority for a South Asian person to access the video following signposting or referral? Do you think it would help a South Asian person to have specific goals for completing the video? What do you think will help a South Asian person engaged with accessing the video?
Intentions: Do you think people of a South Asian background with Rheumatoid Arthritis would be likely to follow the manual that is designed to support them. Is it something they would be likely to do? Why may they not have the intention to follow the advice given in the manual about staying healthy? Do you think people of a South Asian background would want to act on the recommendations as listed in the manual.	Intentions: Following signposting from the consultant do you think a person of a South Asian background would have the intentions / want to access the video?
Reinforcement: Do you think there are any incentives or rewards within the manual for following the content and/or recommendations?	Reinforcement: What do you think would increase the chances of a South Asian person completing / accessing the video? Are there any other ways the support could be offered to South Asian people to encourage them to complete the video?
Optimism: How optimistic are you that this manual can help people from a South Asian background with Rheumatoid Arthritis (ie, increase awareness and confidence and reduce heart disease risk)	Optimism: How confident or negative are you that a South Asian person will access the video when referred/signposted via clinic?
Memory, Attention, and Decision Process: How easy or difficult do you think it will be for patients from a South Asian background to remember or follow the information included in the manual? Do you think a person of a South Asian background would have the time required to complete the manual?	Memory, Attention, and Decision Process: What factors will determine whether or not people decide to access the video after they have been signposted to it? How can we offer the manual so that people of a South Asian background decide to access the video?
Emotions: How does the content within the manual make you feel? How do you think a person from a South Asian background would feel following this manual? Do you think a person’s feelings at the time (mood, feelings towards the intervention, fatigue) may affect whether they do?	Emotions: Do you think the emotional state of a person may affect their ability to access the video?
Behavioural Regulation: Is the addition of homework helpful for people of a South Asian background with Rheumatoid Arthritis? Is the addition of setting yourself goals helpful for people of a South Asian background with Rheumatoid Arthritis? Is the addition of monitoring your own goal setting helpful for people of a South Asian background with Rheumatoid Arthritis?	Behavioural Regulation: Do you think a South Asian person would monitor whether they have / are taking on board information from the video?
	Environmental Content and Resources (Environmental Constraints): Can you think of circumstances or situations that would encourage a South Asian person from accessing the video? Can you think of circumstances or situations that would discourage a South Asian person from accessing the video? How well equipped do you think a South Asian person is to access the video? (i.e., access to a computer / mobile device / access to online resources).
	Social Influences: Do you think family and/or friends would help or hinder a South Asian person in accessing the video? Are you aware of any ways in which a South Asian person would be willing to access the video?

A pilot interview with one participant aided in refining the final interview guide, incorporating any necessary additional topics based on the participant’s feedback. The main of this group was to help adapt the cognitive-behavioural patient education intervention developed by John et al.^15^

Three sessions were conducted to culturally adapt, assess the adaptability and acceptability of the existing cognitive-behavioural intervention among individuals of South Asian origin. These sessions utilised the principles of experienced-based co-design (EBCD),^15^ enabling participants to provide insights and feedback on the intervention’s content. The EBCD approach facilitated a collaborative process involving healthcare staff and individuals with lived experience of RA to critically evaluate current CVD risk knowledge care pathways in rheumatology.

### Preliminary content adaptation

The TDF has 14 theoretical domains that outline determinants of behaviour (knowledge, skills, social/professional role and identity, beliefs about capabilities, optimism, beliefs about consequences, reinforcement, intentions, goals, memory attention and decision processes, environmental context and resources, social influences, emotion, and behavioural regulation). The TDF was used as it is a useful, flexible framework for the assessment of barriers and targeting resources to influence behaviour change to make sure the interventions put into place were appropriate.

### Iterative content adaptation with patient partners

Session 1 focused on participants’ comprehension of cultural beliefs regarding CVD and RA, awareness of CVD risk factors, reactions to CVD-related information, and lifestyle modifications aligned with culturally influenced illness representations. It also delved into participants’ understanding of cardiovascular functioning, the impact of chronic inflammation on CVD development, and the association between RA and CVD risk. A particular focus remained on what and how key messages could be adapted from John et al^15^ intervention to be imported into the new intervention (**Table 2**).

**Table 2.** Cultural modifications and adaptations to the existing intervention.

**Chapter**	**Existing Cognitive Behavioural Intervention**	**Adaptations required for new content**	**Adaptations and content of the final tailored intervention to specific behaviour change techniques**
Chapter 1: What is RA?	*What RA is* was not included as the existing intervention began with what is heart disease?	Spoken in South Asian language. Introduction of RA. Mention of genetics. Cultural beliefs.	Capability for behaviour change - providing knowledge about RA and the importance of looking after your health to ease symptoms.
Chapter 2: How does RA affect the heart?	*What is heart disease* including visuals: - Heart arteries becoming blocked. -Narrowing of arteritis with plaque. Different stages of heart disease. What causes furring up or narrowing of the blood vessels. How RA affects the heart. Things to help prevent heart disease, the text read “a healthy diet should have plenty of fruit, vegetables and wholegrain, (such as brown bread, whole meal pasta or rice, couscous) and be low in fat (butter, ice-cream, full fat cheese, red meat, biscuits, cake doughnuts, chips”.	Spoken in South Asian language. Additional visuals: - Common symptoms of heart disease. -Factors used to calculate heart risk. -Heart disease prevention. Things to prevent heart disease including replacement of foods applicable to those of a South Asian origin: -Whole meal pasta and rice replaced with multi grain chapatti flour. -Butter replaced with ghee. -Biscuits, cake, doughnuts replaced with traditional sweet offerings (mithai ).	Capability: for behaviour change – providing the information on consequences of behaviour and the preventative measures to reduce CVD risk if the right steps are taken.
Chapter 3: Diet	What is my actual risk of heart disease? Example visuals: - White British man smoking and drinking beer. -White British couple.	Spoken in South Asian language. Introduction of a ‘South Asian’ eat well plate. Visuals to include South Asian people. Addition and visuals of South Asian foods were included. Salt and sugar daily recommended intake including demonstration. Suggestions for unhealthy foods to swap for healthy foods. Addition of a role play: Healthy Alternative Swaps. The role play focused on a ‘dinner party’ setting with a discussion between two friends about the decision between healthy vs. unhealthy foods and snacks. Portion control, including demonstration with two different size plates (1 big and 1 small).	Opportunity for behaviour change Behaviour substitution: - Alternative swaps and recommendations given to eat a healthier diet. Techniques demonstrated: -Try using a smaller plate size when having your breakfast, lunch or dinner. -Halving traditional sweet treats to reduce sugar intake. Use of visuals demonstrating how much sugar is in mithai (traditional sweets).
Chapter 4: So what?	Homework tasks What factors need to be changed that are applicable to me	Preventative measures that can be done to reduce CVD risk. Addition of a role play: Unwillingness to Change. The role play demonstrated a discussion between two friends with the focus on a person who was unwilling to change despite being advised by the doctor to make changes. The role play is designed to encourage people to think about their own unhealthy behaviours and find ways to change them and highlight that change does not happen overnight and is a gradual process.	Opportunity for behaviour change by reflecting and having beliefs about your own capability to implement a new behavioural change.
Chapter 5: Now what?	Goal setting and maintaining new behaviour changes	Task given to write down the things that you can change to improve your health. Achieve one goal / task then move onto another. Involve family and/or friends for encouragement.	Motivation for behaviour change – encouraging the use of goal setting and involving those around you (including family and friends) to provide social support to achieve anticipated goals to encourage new habit formation. Rewards and incentive to celebrate achievements.

Session 2 involved a review of the intervention’s content and delivery and gathered suggestions for its adaptation (**Table 2**). Participants’ ideas on the intent to reduce CVD risk were explored by examining their commitment to adopting specific behaviours, considering the pressures and barriers hindering behaviour change, and reflecting on past experiences and anticipated obstacles. This session involved engagement with patient partners, whose insights are relevant to include.

**Figure 1.**
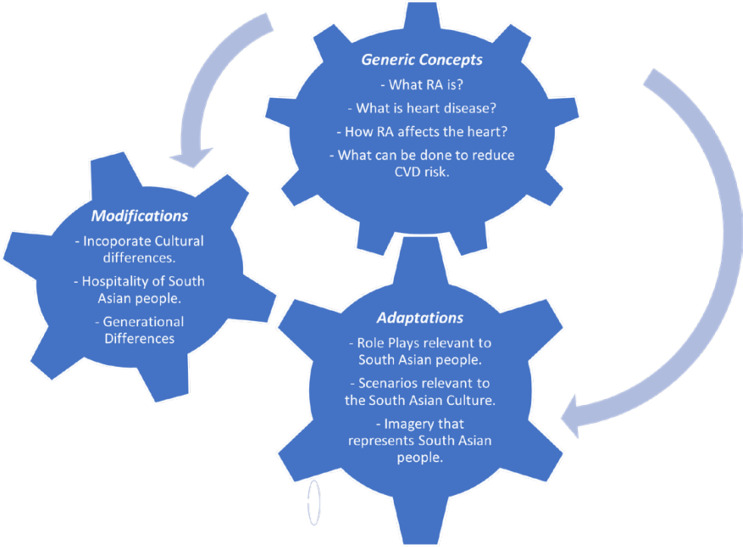
Components of the pre-existing cognitive behavioural model representing generic information relevant to the new intervention along with the modifications and adaptations that were required.

Session 3 focused on obtaining final feedback from patient partners on the newly adapted content. Particular attention paid to messages, visualisation used and its relation to cultural practices (**Figure 1**).

### Analysis

Data gathered from the sessions were analysed independently by RT and KK using a thematic framework. Establishing similar patterns during the discussions was undertaken by RT and KK by listening to the audio recordings. In general, the coding of the audio recordings was similar between the two researchers. Further review of the coding of sessions 1 and 2 was conducted by AK and SG to ensure accurate interpretations of the coded data. The results from sessions 1 and 2 enabled us to identify the key components of the existing cognitive behavioural intervention that required adapting and modifying for people of South Asian origin.

## RESULTS

**Figure 1** shows the three overarching themes identified relating to the adaptation and development of the new cognitive behavioural intervention: (1) general attitudes and beliefs about CVD risk, (2) modifications to incorporate cultural influence and (3) adaptations required to respond to potential barriers.

### General and specific attitudes and beliefs towards CVD risk

Participants generally responded positively to the existing cognitive-behavioural intervention. There were commonalities in knowledge regarding RA, CVD, the impact of RA on the heart, and strategies to reduce CVD risk between South Asian and White British cultures. However, patient partners noted the need for adaptations in language tone, addressing perceived ill health among South Asians, incorporating culturally relevant aspects related to diet, exercise, myths, generational behaviours, family/societal influence, and the inclusion of appropriate visuals specifically targeting individuals of South Asian origin (Quote 1). While the content related to RA and CVD was deemed generic, these cultural-specific modifications were identified as necessary for audience alignment.

#### Example of patient partner quotes:

Quote 1: *Manual is perfect, needs tweaking for the right audience.* [Male] (All quotes in **Table 3**)

**Table 3.** Patient Partner quotes.

**Quote:**	**Patient Partner Response**

	**General and specific attitudes and beliefs towards CVD risk**
1	Manual is perfect, needs tweaking for the right audience. [Female]
2	Need to be told to fix it. [Female]
3	*Unless you are informed you will not know [Female]*
4	*If I know what can happen, what I need to do to fix it. [Female]*
5	*Can I reverse it if I did all those things. [Female]*
6	*I need to do a few walks, add some salads, control portion sizes etc. [Female]*

	**Modifications to include cultural influence**
7	*I am your typical Asian…I don’t know now how to eat healthy. [Female]*
8	*The idea of eating vegetables is to soak in oil. [Female]*
9	*I can’t eat that. There’s nothing on the menu except for the salad…no there’s nothing, I can’t think of one thing on the menu. [Female]*

	**Adaptations required to respond to barriers**
10	*Images to be used in the new intervention needed to be relatable. [Male]*
11	*Someone who looks like me. [Female]*
12	*I don’t drink beer this doesn’t relate to me. [Female]*
13	*Someone putting a samosa in their mouth…typical fried food. [Male]*

Patient partners highlighted various aspects during the discussions: 1) limitations in current healthcare services and consultations, 2) attributing CVD cause to RA rather than personal behaviours, and 3) recognising the importance of the intervention. Participants expressed unawareness of the elevated CVD risk associated with RA diagnosis and emphasised the need for guidance in managing this problem in the intervention (Quote 2). Limited time during routine consultations was identified as a barrier to receiving information about the link between CVD and RA, leading to concerns regarding the management of a condition that they may not be aware of (Quote 3). Understanding that CVD risks connected to RA, participants emphasised the significance of this knowledge in attributing the cause appropriately, highlighting the value of the existing cognitive-behavioural intervention. This led patient partners to think about their own CVD risk factors. After holding the discussion with patient partners about the increased CVD risk participants shared a willingness to learn and expressed the intervention should break down the CVD awareness for the South Asian patients and what they could do to reduce their risk (Quote 4) and what they could do to reduce their risk (Quote 5). One participant mentioned, more needs to be discussed on exercising and how people of South Asian origin view food portions (Quote 6). There were opportunities to understanding the link between RA and increased CVD risk if framed correctly in the intervention it will allow audiences to reflect on the lifestyle and may encourage behavioural changes to reduce CVD risk.

#### Example of patient partner quotes:

Quote 2: *Need to be told to fix it.* [Female]

Quote 3: *Unless you are informed you will not know* [Female]

Quote 4**:** *If I know what can happen, what I need to do to fix it.* [Female]

Quote 5**:** *Can I reverse it if I did all those things.* [Female]

Quote 6**:** *I need to do a few walks, add some salads, control portion sizes etc.* [Female]

#### Modifications to include cultural influence

Participants spoke of the modifications that would be required to incorporate culture, and how approaches to hospitality could be adapted, and generational differences to the existing intervention. These changes include behaviour change techniques such as providing information about health consequences using accessible content to increase knowledge and awareness of CVD risk and RA. These changes would be presented in a more accessible format by targeting specific beliefs of people of South Asian origin. The group expressed particular attention to the following: language, metaphors, content, concepts, goals, and context to enhance relevance, acceptability, and comprehensibility. The name of the intervention Meri Sehat, Mere Rules (My health, My Rules) was also decided by the group.

From the interview data, it was apparent that more awareness on the healthy consumption of food needed to be incorporated within the intervention. A paucity of awareness about counting calories was observed. Being aware of calorie consumption and plate size also was less in the South Asians and the participants requested this concept also to be incorporated in the intervention. In particular, the South Asian food industry has less labelling on highly calorific foods. One participant spoke of not knowing how to eat healthily (Quote 7, 8). Within South Asian culture food plays a pivotal part in everyday life for many. Participants spoke about family gatherings and functions where there is plentiful food. More importantly, the weddings, functions and party catering mind-set has not changed much over the past six decades. South Asian diets are generally healthy, however, foods served at family gatherings and functions can be quite a calorie dense. One participant spoke about attending a wedding with limited healthy options (Quote 9). This is an important implication for the newly developed content. Example of patient partner quotes:

Quote 7**:** *I am your typical Asian…I don’t know now how to eat healthy.* [Female]

Quote 8**:** *The idea of eating vegetables is to soak in oil.* [Female]

Quote 9*: I can’t eat that. There’s nothing on the menu except for the salad…no there’s nothing, I can’t think of one thing on the menu.* [Female]

Another recurring theme within the interview data was the generational gap. Participants spoke about the older generation might find it harder to adapt to calorie count since they might have approached food consumption embedded to historical traditions. A key component for the newly developed content is how to address scenarios where people are unwilling to change.

### Adaptations required to respond to barriers

Motivational drivers specific to individuals of South Asian origin were essential in shaping the intervention. The proposed approach involved an online educational video as the preferred mode of delivery, to be hosted on platforms such as the NRAS website (www.nras.org.uk/apnijung), ensuring broader accessibility for patients and clinicians at the national and international levels. Language emerged as a barrier for those of South Asian origin, highlighting the need for content that is understandable to individuals with limited English proficiency. Verbal content presented in video format was deemed more accessible, particularly for those whose first language is not English.

Session three emphasised the importance of relatability. Participants expressed the need for visuals that are relatable, suggesting changes in images to resonate with the target audience (Quote 10). Featuring individuals from a South Asian background in the videos was considered an effective approach for connecting with the viewers (Quote 11). Notably, the existing intervention primarily catered to and included visuals relevant to the White British population, including foods associated with the Western English diet (Quote 12). Cultural differences, particularly regarding food, were emphasised, and appropriate adaptations with culturally specific visuals became paramount for developing the new content (Quote 13) ensuring cultural appropriateness for individuals of South Asian origin. Patient partners emphasised the message that the consumption of cultural foods should be controlled rather than eliminated, highlighting the importance of this concept in the intervention.

Example of patient partner quotes:

Quote 10**:** *Images to be used in the new intervention needed to be relatable.* [Male]

Quote 11**:** *Someone who looks like me.* [Female]

Quote 12**:** *I don’t drink beer this doesn’t relate to me.* [Female]

Quote 13**:** *Someone putting a samosa in their mouth… typical fried food.* [Male]

Finalised adaptation with patient partners and feedback Together with the information gathered from sessions 1 and 2 this third session was dedicated to organising content into sections and verifying. Here, a script for the new intervention was developed. RT, KK, SG, and AK, discussed the identified themes to create new content for the intervention. The script was then reviewed by the wider research team for further input. The development of the script and video involved the entire multidisciplinary and interdisciplinary team, ensuring that all key concepts were appropriately addressed while considering South Asian culture and traditions. The final script was shared with patient partners for their agreement.

The newly developed and adapted intervention was divided into five sections: (1) explaining RA, (2) discussing how RA affects the heart (including patient role play), (3) focusing on portion control and keeping a food diary as part of the diet (including patient role play), (4) exploring the reasons for making lifestyle changes, and (5) setting goals for the future (including patient role play). Each section was delivered in South Asian language and incorporated interactive elements to engage the audience. Visual pictures depicting culturally traditional healthy and unhealthy foods were included in each section. Role plays were employed to convey messages related to managing healthy food choices in real-life scenarios and dispelling myths about lifestyle choices. Patient partners actively contributed to the role plays, incorporating key cultural and traditional practices related to food use. Participants were given the opportunity to verify the adapted content, and certain wordings were modified to enhance message delivery.

## DISCUSSION

This study represents the first culturally adapted cardiovascular disease (CVD) intervention specifically designed for non-English speaking South Asian individuals living with RA. By involving patient partners and employing the ecological validity model.^12^ we successfully developed a culturally tailored CVD intervention to facilitate behavioural change. Our approach aligns with other studies that emphasise the importance of retaining essential components, such as language tone and cultural considerations, in tailored interventions.^16^ Furthermore, our findings provide valuable insights into the barriers to behavioural change, acceptability, feasibility, and potential content for an online intervention targeting non-English speaking South Asian individuals with RA.

Our study emphasises the importance of a comprehensive framework and meticulous process for cultural adaptation, going beyond mere translation. Our approach involved capturing and integrating core cultural factors, utilising metaphors, characters, and illustrations that resonate with the target population’s cultural context. Notably, the application of health psychology theory proved effective in understanding the development of CVD content based on patient partners’ perspectives. These findings contribute to advancing cultural adaptations and highlight the significance of incorporating theoretical frameworks in understanding and addressing health-related^17^ issues in South Asian groups. Successful cultural adaptation necessitates a collaborative effort between team experts and patient partners, as demonstrated in this study. Balancing the insights and perspectives from both groups is crucial. Patient partners living with RA played a pivotal role in informing the content development and establishing cultural relevance, while experts provided valuable input regarding clinical significance. In this project, we employed an interdisciplinary approach, ensuring the inclusion of viewpoints from general practitioners and secondary care clinicians, which further enriched the message delivery.

The dynamics of food, family, and community support highlighted by our patient partners are vital motivators for promoting healthy behaviours among individuals of South Asian origin. Studies indicate that active family involvement in diabetes management leads to improved management plans.^18^ Our patient partners emphasised that while the overall South Asian diet is healthy, temptations arising from social gatherings such as weddings and religious events often limit the availability of healthier food choices. Furthermore, concerns were expressed regarding the lack of regulation and labelling of certain South Asian foods, such as mithai (traditional sweets), resulting in insufficient awareness of their calorie content. The food industry must take responsibility by incorporating innovative and revised South Asian food recipes to promote a healthier lifestyle. Collaborating with organisations like the British Heart Foundation and the South Asian Health Foundation, which provide resources for CVD and diabetes awareness.^17^ can enhance our Intervention and facilitate its integration within health settings and the wider South Asian community.

The study demonstrates strengths in the development of an effective culturally adapted CVD educational video for non-English speaking South Asians through collaboration with patient partners and an interdisciplinary team. The intervention will be made accessible nationally and internationally through NRAS. A limitation of the study is the small size of the patient partner group, which may have limited the variability of perspectives. Including a larger and more diverse group, including community leaders and representatives from the food industry, could have provided additional insights into traditional cultural practices and food labelling dynamics. However, the patient partner group provided valuable ideas that contribute to the wider South Asian culture.

## CONCLUSIONS

This cultural adaptation study serves as a model for future adaptations in chronic conditions, emphasising the interdisciplinary approach and collaboration among stakeholders. The intervention will be accessible worldwide through www.nras.org.uk/apnijung, enabling tailored interventions for individuals of South Asian origin. We urge primary care and rheumatology clinicians to integrate this intervention into annual review clinics and for those at risk of CVD. The next phase should involve evaluating the intervention’s effectiveness, focusing on clinical outcomes related to CVD and measuring patient lifestyle changes.

## DECLARATION

KK has been awarded grants by Global Pfizer and has received honorarium from UCB and AbbVie for presenting talks.
